# Abnormal white matter structural network topological property in patients with temporal lobe epilepsy

**DOI:** 10.1111/cns.14414

**Published:** 2023-08-25

**Authors:** Haiming Ai, Chunlan Yang, Min Lu, Jiechuan Ren, Zhimei Li, Yining Zhang

**Affiliations:** ^1^ Faculty of Science and Technology Beijing Open University Beijing China; ^2^ College of Life Science and Bioengineering Beijing University of Technology Beijing China; ^3^ Beijing Tiantan Hospital Capital Medical University Beijing China; ^4^ Department of Equipment Baoding first Central Hospital Baoding China

**Keywords:** diffusion tensor imaging, graph theory analysis, structural network, temporal lobe epilepsy, white matter

## Abstract

**Background:**

Diffusion tensor imaging (DTI) studies have demonstrated white matter (WM) abnormalities in patients with temporal lobe epilepsy (TLE). However, alterations in the topological properties of the WM structural network in patients with TLE remain unclear. Graph theoretical analysis provides a new perspective for evaluating the connectivity of WM structural networks.

**Methods:**

DTI was used to map the structural networks of 18 patients with TLE (10 males and 8 females) and 29 (17 males and 12 females) age‐ and gender‐matched normal controls (NC). Graph theory was used to analyze the whole‐brain networks and their topological properties between the two groups. Finally, partial correlation analyses were performed on the weighted network properties and clinical characteristics, namely, duration of epilepsy, verbal intelligence quotient (IQ), and performance IQ.

**Results:**

Patients with TLE exhibited reduced global efficiency and increased characteristic path length. A total of 31 regions with nodal efficiency alterations were detected in the fractional anisotropy_ weighted network of the patients. Communication hubs, such as the middle temporal gyrus, right inferior temporal gyrus, left calcarine, and right superior parietal gyrus, were also differently distributed in the patients compared with the NC. Several node regions showed close relationships with duration of epilepsy, verbal IQ, and performance IQ.

**Conclusions:**

Our results demonstrate the disruption of the WM structural network in TLE patients. This study may contribute to the further understanding of the pathological mechanism of TLE.

## 1 INTRODUCTION

Temporal lobe epilepsy (TLE) is regarded as a disease characterized by brain network disruption which may be due to long‐term epileptic discharge in epileptic seizure.^1^ Previous functional studies based on electroencephalography (EEG), functional magnetic resonance imaging (fMRI), and single‐photon emission computed tomography have reported that epileptic patients show aberrant global and local network connectivity. As an advanced neuroimaging technique, fMRI has become a primary effective tool for brain network analysis of TLE.

Diffusion tensor imaging (DTI), which is another commonly used modality of MRI, provides structural information complemented with functional changes derived from fMRI. The anatomical mechanism of TLE is still unclear. Limited previous studies on the white matter (WM) structural network of patients with TLE have reported the aberrant default network,^2^, ^3^ limbic network,^4^ and individual‐level brain network^5^ obtained by using DTI. Liu et al. and Xu et al.^2^, ^6^ further observed decreased global efficiency and widespread reductions in the regional efficiency in the brain areas of the left mesial TLE. Jiang et al.^7^ reported that the disrupted brain network is associated with deficits in the alertness of patients with right TLE. Therefore, changes in the TLE patients' network not only occurred in the temporal lobe but also became widespread in other brain regions, including the default mode network, executive control network, and emotional network. Patients with non‐lesional temporal lobe epilepsy also show cognitive dysfunction, such as attention and memory, which affects patients' executive function.^8^ The changes of WM network structure may play an important role in epileptic seizure and epileptic discharge transmission.

Accordingly, the main research objectives of this paper is to explore the structural connectivity of TLE white matter based on graph theory. We distinguished the abnormal WM in TLE from the perspective of brain topology. The graph‐based theoretical parameters of the brain network properties were analyzed. The relationships between the topological properties and clinical information, namely, duration of epilepsy, verbal intelligence quotient (IQ) and performance IQ, were also explored. The results may elucidate the mechanism of WM structural network disruption in patients with TLE.

## 2 MATERIALS AND METHODS

### 2.1 Participants

Eighteen TLE patients (10 males and 8 females; mean age, 30.4 ± 8.16 years, range = 18–46) were recruited from the Beijing Tiantan Hospital, of which 9 were left‐lateralized (4 females, mean age = 27.8, range = 18–37) and 6 were right‐lateralized (3 females, mean age = 32.6, range = 20–46). Moreover, Twenty‐nine age‐ and gender‐matched normal controls (NC) (17 males and 12 females; mean age, 27.8 ± 5.78 years, range = 21–42) were recruited. The NC reported no history of neurological or psychiatric disorders. Clinical information (i.e., duration of epilepsy, age of seizure onset, EEG data, and MRI data) and the scores of Wechsler Adult Intelligence Scale—Revised for China which are hereby referred to as the verbal IQ and performance IQ,^9^ were collected from all subjects. Each subject signed a written informed consent prior to the study, which was approved by the Ethics Committee of Beijing Tiantan Hospital.

### 2.2 Image acquisition

MRI data were acquired using a 3.0‐T Siemens Trio MRI scanner (Siemens Medical). The parameters of the single‐shot spin‐echo EPI sequence to acquire diffusion‐weighted images were as follows: repetition time (TR) = 8000 ms, echo time (TE) = 60 ms, 30 diffusion gradient directions (*b* = 1000 s/mm^2^), 1 volume without diffusion‐weighted direction (*b* = 0 s/mm^2^), field of view (FOV) = 256 mm × 256 mm, and slice thickness was 2.0 mm. Each volume consisted of 75 axial slices.

T1‐weighted images were acquired using a magnetization‐prepared rapid gradient echo sequence with the following parameters: TR = 2300 ms, TE = 2.32 ms, inversion time (TI) = 900 ms, flip angle = 8°, FOV = 100 mm × 100 mm, resolution matrix = 256 × 256, slice number = 192, and slice thickness = 0.9 mm.

### 2.3 Data preprocessing and WM structural network construction

The main steps of data preprocessing were as follows: (1) file format conversion (from DICOM to NIFTI images); (2) brain mask estimation (b0 image was used to estimate); (3) image cropping and eddy current correction, the diffusion‐weighted images were registered to the b0 image by affine transformation to correct the eddy current distortions and the motion artifacts; (4) averaging of multiple acquisitions, the fslmaths command of FSL were used to average multiple diffusion‐weighted images; (5) calculation of fractional anisotropy (FA). The procedure was performed by the PANDA software (https://www.nitrc.org/projects/panda).^10^ Automated anatomical labeling (AAL) template was used to construct the WM structural network of TLE patients and NC.^11^ In this process, the brain, except the cerebellum, was divided into 90 cortical regions. The brain regions included the primary cortex, association cortex, paralimbic cortex, and subcortical structures.^12^, ^13^ Table 1 depicts the names and abbreviations of these brain regions.

**TABLE 1:** Names and abbreviations of AAL brain regions.

Index	Regions	Abbreviation	Index	Regions	Abbreviation
1,2	Precentral gyrus	PreCG	47,48	Lingual gyrus	LING
3,4	Superior frontal gyrus	SFGdor	49,50	Superior occipital gyrus	SOG
5,6	Superior orbitofrontal gyrus	ORBsup	51,52	Middle occipital gyrus	MOG
7.8	Middle frontal gyrus	MFG	53,54	Inferior occipital gyrus	IOG
9,10	Middle orbitofrontal gyrus	ORBmid	55,56	Fusiform gyrus	FFG
11,12	Inferior frontal gyrus (opercular)	IFGoperc	57,58	Postcentral gyrus	PoCG
13,14	Inferior frontal gyrus (triangular)	IFGtriang	59,60	Superior parietal gyrus	SPG
15,16	Inferior orbitofrontal gyrus	ORBinf	61,62	Inferior parietal gyrus	IPL
17,18	Rolandic operculum	ROL	63,64	Supramarginal gyrus	SMG
19,20	Supplementary motor area	SMA	65,66	Angular gyrus	ANG
21,22	Olfactory gyrus	OLF	67,68	Precuneus	PCUN
23,24	Medial frontal gyrus	SFGmed	69,70	Paracentral lobule	PCL
25,26	Medial orbitofrontal gyrus	ORBmed	71,72	Caudate nucleus	CAU
27,28	Rectus gyrus	REC	73,74	Lenticular nucleus, putamen	PUT
29,30	Insula	INS	75,76	Lenticular nucleus, pallidum	PAL
31,32	Anterior cingulate gyrus	ACG	77,78	Thalamus	THA
33,34	Middle cingulate gyrus	MCG	79,80	Heschl gyrus	HES
35,36	Posterior cingulate gyrus	PCG	81,82	Superior temporal gyrus	STG
37,38	Hippocampus	HIP	83,84	Temporal pole:superior temporal pole	TPOsup
39,40	Parahippocampal gyrus	PHG	85,86	Middle temporal gyrus	MTG
41,42	Amygdala	AMYG	87,88	Temporal pole:middle temporal pole	TPOmid
43,44	Calcarine	CAL	89,90	Inferior temporal gyrus	ITG
45,46	Cuneus	CUN			

Abbreviation: AAL, automated anatomical labeling.

The basic network elements comprise nodes and edges.^10^, ^14^ The primary steps of weighted WM network construction were as follows: (1) affine transformation of FA image to its corresponding T1‐weighted image; (2) registration of affinely registered images to the Montreal Neurological Institute (MNI) space; (3) inverse transformation of images from the MNI space back to the individual space; (4) tracking of WM fibers based on a deterministic tracking algorithm (tracking stopped when FA <0.2 or tracking turning angle >45°)^6^, ^15^; (5) construction of WM networks matrix based on the indexed by values of FA. For FA_weighted network, the network edges were defined by value of FA >0.2, for the binary network, the network edges were defined as 1 if FA between the two regions was larger than the threshold (FA >0.2) and as 0 otherwise. Two WM structural network matrices (90 × 90) with FA_weighted (FA_wei) and FA binary (FA_bin) were obtained for each participant.

### 2.4 Graph theoretical analysis

Graph theoretical analysis was performed to detect differences in the WM structure network parameters between the TLE and NC groups. The global network properties, regional network properties, and small‐world property of WM structural network were calculated using the GRETNA software (http://www.nitrc.org/projects/gretna/).^16^ Brain Connectivity Toolbox (https://sites.google.com/site/bctnet/) was used to calculate the property of degree to determine the hub node.^17^

#### 2.4.1 Small‐world property

Small‐world property [sigma (*σ*)] is a feature of a network with high clustering coefficient and low characteristic path length.^18^ The network showed small‐world property if *σ* > 1. A high value of *σ* indicates a strong small‐world property of network.^19^ This property is calculated follows:

σ=γλ=CCrandLLrand

where *C*_*rand*_ is the mean weighted clustering coefficient of random networks and *L*_*rand*_ is the mean weighted characteristic path length of random networks.

#### 2.4.2 Global parameters

1. Global efficiency (*E*_*g*_) reflects the efficiency of information exchanged across the network. High efficiency indicates low cost.^17^, ^20^ *E*_*g*_ is calculated as follows:
$$E_{g}=\frac{1}{n}\sum_{i\in N}E_{i}=\frac{1}{n}\sum_{i\in N}\frac{\sum_{i\in N,j\neq i}d_{\mathrm{ij}}^{-1}}{n-1}$$

where *E*_*i*_ is the efficiency of node *i* and *d*_*ij*_ is the shortest path length between nodes of *i* and *j*.

2 Clustering coefficient (*C*_*p*_) describes the tightness of connections between nodes,^17^ and is calculated as follows:
$$C_{p}=\frac{1}{n}\sum_{i\in N}C_{i}=\frac{1}{n}\sum_{i\in N}\frac{2t_{i}}{k_{i}\left(k_{i}-1\right)}$$

where *C*_*i*_ is the clustering coefficient of node *i*, *t*_*i*_ is the number of triangles around a node *i*, and *k*_*i*_ is the degree of the node.

3 Local efficiency (*E*_*loc*_) is defined as the average efficiency of the local subgraphs and is calculated as follows^17^, ^20^:
$$E_{\mathrm{loc}}=\frac{1}{n}\sum_{i\in N}E_{\mathrm{loc},j}=\frac{1}{n}\sum_{i\in N}\frac{\sum_{j,h\in N,j\neq i}a_{\mathrm{ij}}a_{\mathrm{ih}}{\left[d_{\mathrm{jh}}\left(N_{i}\right)\right]}^{-1}}{k_{i}\left(k_{i}-1\right)}$$

in the neighborhood of node *i*, *a*_*ij*_ is the connection status between *i* and *j*, *d*_*jh*_ (*N*_*i*_) is the length of the shortest path between nodes of *j* and *h*, and *k*_*i*_ is the degree of node *i*.

4 Characteristic path length (*L*_*p*_) describes the optimal path for the information transmission from one node to another node in the network,^17^ and is calculated as follows:
$$L_{p}=\frac{1}{n}\sum_{i\in N}L_{i}=\frac{1}{n}\sum_{i\in N}\frac{\sum_{j\in N,j\neq i}d_{\mathrm{ij}}}{n-1}$$

where *d*_*ij*_ is the shortest path length between nodes of *i* and *j*.

#### 2.4.3 Regional parameters

1. Nodal efficiency (*E*_*nodal*_) is defined as the inverse of the harmonic mean of the minimum path length between the index node and all other nodes in the network.^21^ High nodal efficiency indicates high information transmission speed between nodes in the network. *E*_*nodal*_ is calculated as follows:
$$E_{\text{nodal}}\left(i\right)=\frac{1}{\left(n-1\right)}\sum_{j\in N}\frac{1}{L_{i,j}}$$

where *L*_*i*,*j*_ is the length of the shortest path between nodes of *i* and *j*.

2 Hubs substantially contribute to the whole‐brain function. The indicators of hubs include degree and centrality.^22^ The degree describes the number of edges directly connected to a node and is calculated as follows^19^:
$$k_{i}=\sum_{j\in N}a_{\mathrm{ij}}$$

where *a*_*ij*_ is the connection status between nodes of *i* and *j*. The value will be 1 if a link exists, and 0 if otherwise.

### 2.5 Statistical analysis

Two‐sample *t* test was performed with the influence of age and gender controlled to determine whether significant differences exist in the topological property (*E*_*g*_, *C*_*p*_, *E*_*loc*_, *L*_*p*_, *σ*, and *E*_*nodal*_) of the WM structural networks between the two groups, and GRETNA software was used for statistical calculation.^16^

Partial correlation analysis was performed to explore the correlation between network topological properties and the three clinical characteristics (duration of epilepsy, verbal IQ, and performance IQ) of the TLE patients. Age and gender were considered covariates of no interest. Aside from age and gender, age at seizure onset was also considered to analyze the correlation between nodal efficiency and duration of epilepsy. Multiple comparison correction was applied on the comparison and correlation results by using false discovery rate (FDR) at *p* = 0.05.^23^ IBM SPSS Statistics 25 was used for partial correlation analysis and Matlab 2015a was used for FDR correction.

## 3 RESULTS

Binary and weighted networks for the subjects were constructed. The BrainNet Viewer software was used to display the network hubs (http://www.nitrc.org/projects/bnv/).^24^ Differences in the topological parameters between the TLE and NC groups were detected. Given that the binarization of the network may result in the partial loss of information, only the weighted networks were considered in the analysis of the correlation between network topological properties and the three clinical characteristics.

### 3.1 Participants

No significant difference was observed in the age and gender between the TLE and NC groups (*p* > 0.05). However, the scores of verbal IQ in the TLE group were lower than those of the NC group. Similarly, the scores of performance IQ in the TLE group were also lower than those of the NC group. In addition, a significant negative correlation was noted between the duration of epilepsy and age at seizure onset (*r* = −0.632, *p* < 0.05). Table 2 presents the demographics and clinical characteristics of subjects.

**TABLE 2:** Demographics and clinical characteristics of subjects.

Characteristics	Variables (mean ± SD)		*p‐*Value
	Patients (*N* = 18)	Controls (*N* = 29)	
Age (years)	30.4 ± 8.16	27.8 ± 5.78	0.198
Gender (male/female)	10/8	17/12	0.836
Duration of epilepsy (years)	13.90 ± 7.15	–	–
Age at seizure onset	16.28 ± 10.69	–	–
Verbal IQ	90.00 ± 11.57	109.45 ± 11.91	<0.001*
Performance IQ	95.06 ± 10.90	109.00 ± 10.51	<0.001*

Abbreviations: IQ, intelligence quotient; SD, standard deviation.

* *p* < 0.001.

### 3.2 Network analysis

#### 3.2.1 Small‐world property of WM structural networks

All the NC and TLE patients showed small‐world properties in both binary and weighted networks (*σ* > 1). Compared with the NC group, *σ* increased in the TLE group, but there was no significant difference between the groups (*p* > 0.05).

#### 3.2.2 Global parameters of WM structural networks

**FIGURE 1:**
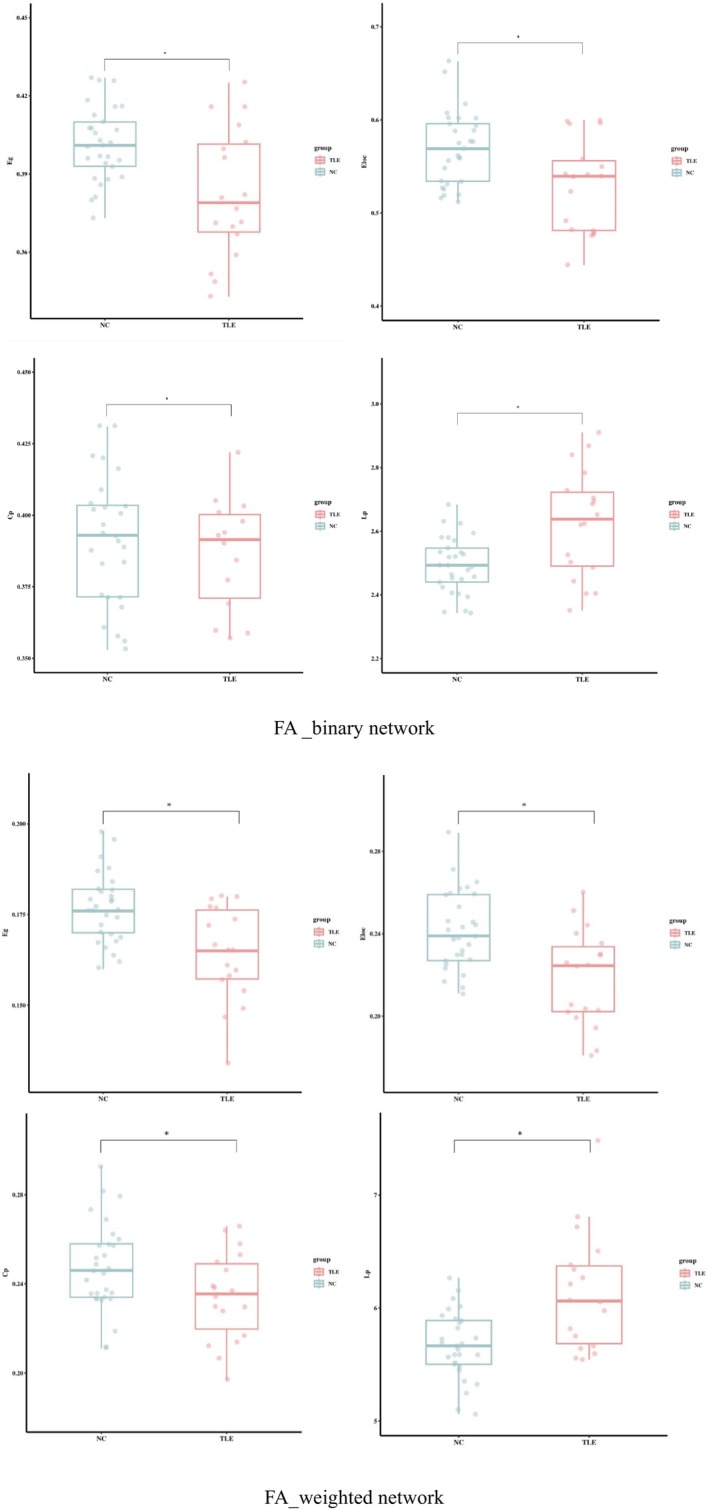
Histogram of the statistical differences in the network topological properties. *p < 0.05, FDR corrected. Abbreviations: FA, fractional anisotropy; NC, normal controls; TLE, temporal lobe epilepsy.

Figure 1 showed the differences in the global parameters for the binary and weighted WM structural networks between the two groups, and plotted by R 4.3.1 software. The Kolmogorov–Smirnov test showed that the topological properties between the two groups, *p* > 0.05, followed the normal distribution. Compared with the NC group, significantly reduced *E*_*g*_, *E*_*loc*_ and *C*_*p*_, but increased *L*_*p*_ were detected in the TLE group (*p* < 0.05, FDR corrected).

#### 3.2.3 Regional parameters of WM structural networks

##### Nodal efficiency

Nodal efficiency (*E*_*nodal*_) measures the average shortest path between a given node and other nodes in the network. In the FA_weighted network, the two‐sample *t* test results showed that compared with the NC group, *E*_*nodal*_ of 31 nodes in the TLE group were different (*p* < 0.05, FDR corrected). The results are listed in Table 3.

**TABLE 3:** Regions with different nodal efficiencies in the FA_weighted network between TLE patients and NC.

Regions	*p*‐Value	Regions	*P*‐Value	Regions	*p*‐Value
Central region		Frontal lobe		Parietal lobe	
PreCG_L	0.024*	SFGdor_R	0.024*	SMG_L	0.032*
ROL_R	0.024*	ORBsup_L	0.024*	ANG_L	0.047*
PoCG_L	0.046*	MCG_L	0.024*	Temporal lobe	
ROL_L	0.049*	REC_L	0.033*	MTG_L	0.024*
Limbic lobe		IFGoperc_R	0.038*	STG_L	0.047*
ACG_R	0.024*	ORBmed_R	0.038*	ITG_L	0.047*
PCG_L	0.024*	PCL_L	0.046*	Occipital lobe	
PHG_L	0.024*	ORBinf_L	0.047*	IPL_L	0.026*
TPOmid_L	0.024*	SFGdor_L	0.047*	IPL_R	0.026*
TPOsup_L	0.024*	OLF_R	0.049*	LING_L	0.043*
PCG_R	0.038*	Insula			
PHG_R	0.040*	AMYG_L	0.024*	CAU_L	0.047*

*Note*: Classification of brain regions was based on AAL proposed by Tzourio‐Mazoyer et al.^11^

Abbreviations: AAL, automated anatomical labeling; FA, fractional anisotropy; NC, normal controls; TLE, temporal lobe epilepsy.

* *p* < 0.05, FDR correct.

##### Hub regions

**FIGURE 2:**
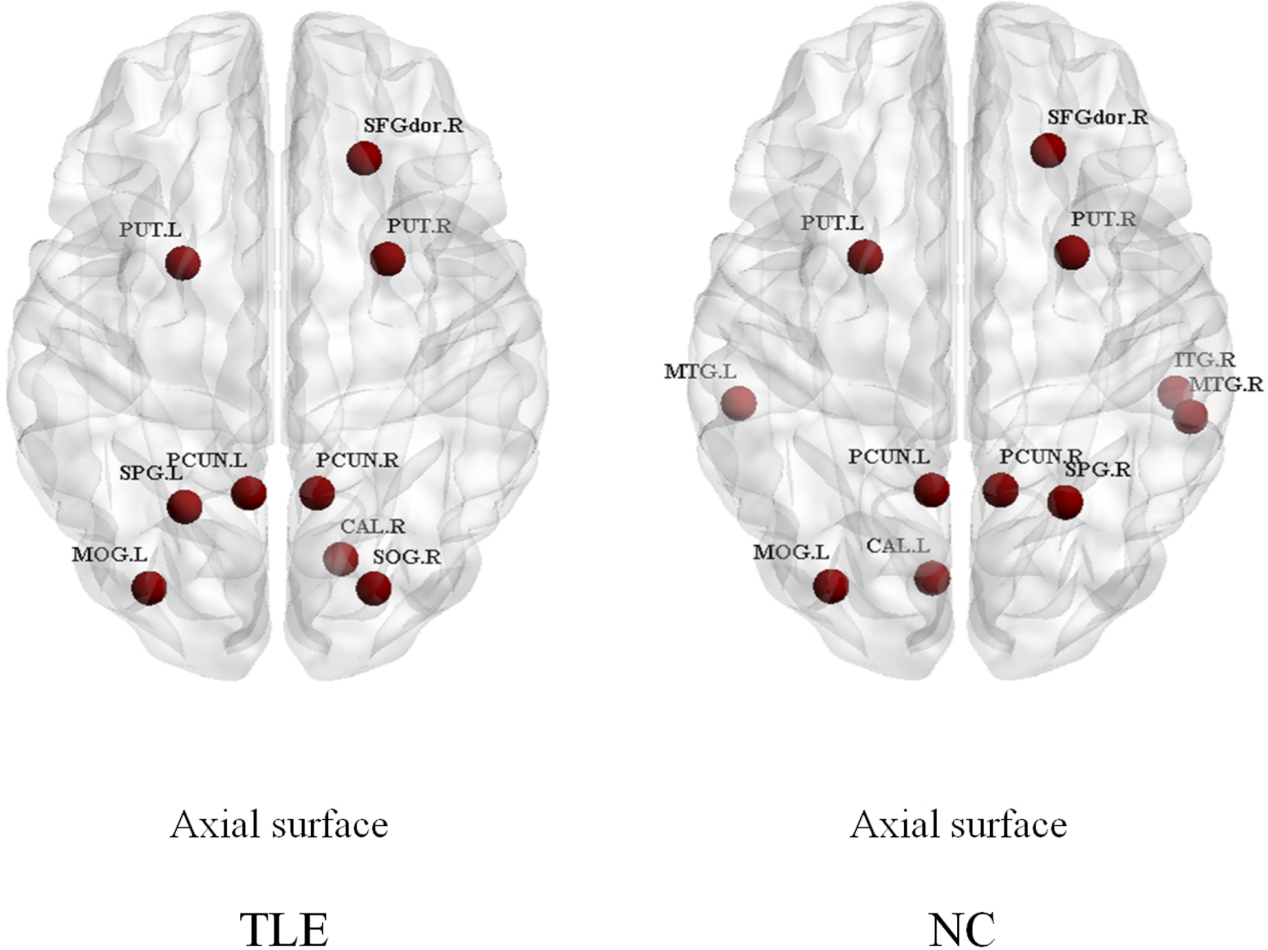
Hub regions detected in groups of TLE and NC. Hubs were illustrated as red circles in the TLE and NC groups. Abbreviations: NC, normal controls; TLE, temporal lobe epilepsy.

In this study, the mean degree of the TLE group was 9.088, whereas that of the NC group was 9.782. The results showed that the shared hubs spread in the association cortices. However, the hubs of TLE patients were outside the temporal lobe regions. The hubs were visualized in Figure 2, and the hubs are listed in Table 4. The whole‐brain degree of TLE and NC groups were drawn in the Figures S1 and S2.

**TABLE 4:** Hub regions in the TLE and NC groups.

Hub Regions	NC group	Degree	TLE group	Degree	Class
Parietal lobe	PCUN_R	14.966	PCUN_R	14.278	Association
	PCUN_L	13.621	PCUN_L	12.722	Association
	SPG_R	10.379	SPG_L	9.333	Association
Occipital lobe	MOG_L	10.414	MOG_L	9.278	Association
	CAL_L	10.138	CAL_R	9.500	Primary
			SOG_R	9.722	Association
Limbic lobe	PUT_R	13.448	PUT_R	12.500	Subcortical
	PUT_L	14.276	PUT_L	14.000	Subcortical
Frontal lobe	SFGdor_R	10.483	SFGdor_R	9.167	Association
Temporal lobe	MTG_L	10.172	–	–	Association
	MTG_R	10.069	–	–	Association
	ITG_R	10.138	–	–	Association

Abbreviations: NC, normal controls; TLE, temporal lobe epilepsy.

#### 3.2.4 Correlation between the network topological properties and clinical characteristics

Partial correlation analyses of the FA_weighted WM structural network properties of *σ*, *E*_*g*_, *E*_*loc*_, *C*_*p*_, *L*_*p*_, and *E*_*nodal*_ to the clinical characteristics were performed for patients, with corrections for age and gender.

No correlation was found between the global network properties and clinical characteristics. However, the regional parameter of *E*_*nodal*_ (nodal efficiency) demonstrated an effective capability to detect correlation with the clinical characteristics. The *E*_*nodal*_ in several regions showed negative correlations with the duration of epilepsy, but showed positive correlations with the verbal IQ and performance IQ in other regions. The detailed results are given in Table 5.

**TABLE 5:** Partial correlation between the nodal efficiency and clinical characteristics in the FA_weighted network.

Node	Duration of epilepsy		Verbal IQ		Performance IQ	
	*r*	*p*‐Value	*r*	*p*‐Value	*r*	*p*‐Value
INS_L	−0.519	0.048*	—	—	—	—
HIP_L	−0.548	0.034*	—	—	—	—
SOG_L	−0.697	0.004**	—	—	—	—
SOG_R	−0.751	0.001**	—	—	—	—
IOG_L	−0.643	0.01*	—	—	—	—
SMG_L	−0.531	0.042*	—	—	—	—
TPOsup_L	−0.713	0.003**	—	—	—	—
TPOmid_L	−0.659	0.008**	—	—	—	—
OLF_L	—	—	0.509	0.044*	—	—
PreCG_R	—	—	—	—	0.501	0.048*
OLF_R	—	—	—	—	0.523	0.038*
IOG_R	—	—	—	—	0.619	0.011*
PoCG_L	—	—	—	—	0.607	0.013*
ANG_R	—	—	—	—	0.558	0.025*

Abbreviations: FA, fractional anisotropy; IQ, intelligence quotient.

* *p* < 0.05

** *p* < 0.01.

## 4 DISCUSSION

We constructed and compared the properties of the brain WM structural networks between patients with TLE and NC. The relationships between the network topological properties and clinical characteristics were analyzed. Compared with the NC, the global efficiency and cluster coefficient decreased, but the characteristic path length increased in the TLE patients. In addition, the nodal efficiencies in the FA_weighted network showed widespread difference between the two groups in the whole brain. The hub regions were absent in the temporal lobe in the TLE patients and shared several hubs in the NC as distributed in the association cortex. Finally, the nodal efficiency of certain node regions in FA_weighted brain network showed negative correlations with the duration of epilepsy. Several node regions showed positive correlations with the verbal IQ and performance IQ.

### 4.1 Network topological properties

#### 4.1.1 Global parameters

Normal brain network was regarded as a small‐world network with high clustering coefficient and short path length.^25^ The small‐world property may be ineffective as sensitive parameter to detect the disruption of brain organization in the early stage of epilepsy. Liu et al.^2^ also believed that both controls and patients with mesial TLE featured small‐world properties. Liao et al.^26^ further explored the changes in small‐world properties in mesial TLE patients.

In our study, the TLE patients showed a significant decrease in the global efficiency and a significant increase in the characteristic path length compared with the NC, these results were consistent with those of previous studies on the brain networks of epilepsy.^5^, ^6^, ^27^, ^28^ Bernhardt et al.^29^ observed increased path length, decreased clustering, and global network efficiency in TLE patients with hippocampal sclerosis, likely reflect topology‐level effects of previously reported diffusion anomalies in multiple long‐range tracts in TLE. They also found that TLE patients suffered more damage to a functional network centered on the hippocampus/parahippocampus. Compared with healthy controls, surface‐based cortical thickness analysis revealed marked atrophy in frontocentral, orbitofrontal, and mesiotemporal regions with TLE patients. This paper is based on simulated structures connectome reorganization, providing a novel perspective for studying the dysfunction of structural governed macroscale dysfunction in TLE.^30^ The decrease in the global efficiency may indicate the impairment of structural connections. Changes in the global network properties suggest a regular small‐world configuration toward a lattice‐like network in patients. The changed network in the patients also showed an increase in the characteristic path length, which reflected the long‐distance connections for information transmission.^2^

Decreased clustering coefficients were found in the TLE group, which was consistent with previous results based on DTI, EEG, and fMRI.^26^, ^27^, ^31^, ^32^ The reduced local efficiency in the TLE patients were consistent with the results of previous studies on the brain network of epilepsy.^6^, ^27^

#### 4.1.2 Regional parameters

The nodal efficiency measures the connectivity of a node to all other nodes in the network and quantifies the importance of the node for communication within the network.^21^ In the FA_weighted network, the regions with decreased nodal efficiency were mainly located in the frontal, limbic, temporal, occipital lobe regions, and insula. Liu et al. and Xu et al.^2^, ^6^ observed that the nodal efficiencies of the left superior temporal gyrus, left temporal pole, middle temporal pole, left inferior temporal gyrus (ITG), left temporal pole (superior temporal), left precentral gyrus, and left rolandic operculum reduced in the TLE group compared with the NC group, which partially agree with our results. The altered nodal efficiency of the bilateral parahippocampal gyrus (PHG) indicated the impairment of memory in TLE patients, which was partially supported by a previous study.^5^ Therefore, we speculated that these alterations were likely caused by the disruption of the brain network in TLE patients.

Hubs play a vital role in the network connection. The majority of hubs in patients and controls were association cortices in our study, which was consistent with the findings of Bernhardt et al.^33^ However, in the group of patients, bilateral middle temporal gyrus (MTG) and right ITG were not hubs in the controls. MTG and ITG were key structures in memory and language processing.^5^ We noted that the nodal efficiency of MTG decreased in the patients with TLE. Xu et al.^6^ also reported the nodal efficiency of right MTG changed in the left mesial TLE patients, which partially supported our results. This result may explain why the hubs of bilateral MTG were absent in the patients. Sone et al.^34^ found that ITG was the hub in the NC group but not in the TLE group, which was partially consistent with our results. Similar to the MTG, the nodal efficiencies of ITG changed in the TLE patients. The superior parietal lobe was also regarded as a hub.^35^ In this study, the hub of the superior parietal gyrus (SPG) in the NC group was located on the right side, but the hub was on the contralateral in the TLE group. SPG may refer to the function of spatial orientation and visual input. Therefore, this hub played an important role in TLE. Accordingly, we hypothesized that epileptic seizures may cause a lateral change in the hubs. Similarly, the left calcarine (CAL) was not a hub in patients but was located in the NC group. However, the right CAL was its hub. This hub may influence the primary visual cortex. To date, limited studies have reported changes in CAL based on fMRI. Zhou et al.^36^ observed decreased functional connectivity with the right dentate nuclei located in the left CAL. Song et al.^37^ noted that the patients with left mesial TLE demonstrated high intrinsic frequency in the CAL. Ridley et al.^38^ reported a degree of reduction in the right‐lateralized focal epilepsies relative to the controls in the left CAL. In addition, the right superior occipital gyrus (SOG) was a hub in the patients but not in the NC. Li et al.^39^ also found increased functional connectivity in the SOG, which may support our result. Several common hubs also appeared in the patients and NC. Precuneus (PCUN) is an important node of the default mode network and participates in episodic memory retrieval.^40^, ^41^ Previous network analysis indicated that the PCUN is a hub of humans.^35^ Ofer et al.^42^ reported that the degree of central hubs, including PCUN, was reduced in seizure‐free TLE. Liao et al.^26^ indicated that the degree of hubs in certain regions, including left PCUN, significantly decreased in TLE. In our results, the degree of PCUN in the TLE group also decreased compared with that in the NC group. The right superior frontal gyrus (SFGdor) was also a hub in both subject groups in our study. However, Wang et al.^5^ observed that the right SFGdor changed in the functional network.

### 4.2 Correlation analysis

The global properties of the network showed no significant correlation with the clinical parameters, which was consistent with previous studies.^5^, ^6^, ^31^

However, the nodal efficiencies of several brain regions were negatively correlated with the duration of epilepsy. The regions are listed in Table 5. Moreover, the nodal efficiencies of the left supramarginal gyrus, left temporal pole, superior temporal pole, left temporal pole, and middle temporal pole were also detected to be changed in the TLE patients. The results about left hippocampus was partially supported by findings of Chiang et al.^1^ Currently, researchers have explored the association between resting‐state functional connectivity and cognition in adult TLE patients.^43^ Cognitive functioning was related to the functional connectivity within the cortex.^44^ Significant differences were found in the activation of brain areas during different tasks between the TLE patients and NC.^45^, ^46^, ^47^ Language networks of people with TLE exhibited fewer structural connections.^48^ In our study, the nodal efficiency of left olfactory gyrus was correlated with verbal IQ. Moreover, several regions with nodal efficiency were related to the performance IQ. Among the regions, the nodal efficiencies of the left postcentral gyrus (PoCG) were also changed in the patients.

Several limitations should also be addressed in our study. First, the sample size of TLE patient group was still small. The larger sample size may be expected to explore more issues such as the lateralization of seizure focus in further subgroup analysis. In addition, more patients were needed to verify the current preliminary results. Second, different definitions of network may influence the results. Further studies using probabilistic tractography should also replicate current results. Third, the WM structural network analysis lacked the complementary evaluation experiments from functional data, such as fMRI or EEG.

## 5 CONCLUSION

In conclusion, graph theory analysis was performed to explore the alterations of the topological properties for the WM structural network of TLE patients. Significant decrease of the global efficiency and increase of characteristic path length were detected in patients. Widespread alterations of the nodal efficiency for the brain network were detected, as well as altered distribution of network hubs. Finally, our results confirmed the correlations between the network properties and the duration of epilepsy, verbal IQ, and performance IQ. These findings support that epilepsy is a network disease, and alterations in the WM structural network may lead to the impairment of IQ.

## CONFLICT OF INTEREST STATEMENT

The authors declare no conflicts of interest.

## Supporting information


Figure S1.
Figure S2.

## Data Availability

The data that support the findings of this study are available from the corresponding author upon reasonable request.
