# Supplementary material for: Abnormal white matter structural network topological property in patients with temporal lobe epilepsy
Source: CNS Neurosci Ther. 2023 Aug 25;30(1):e14414. doi: 10.1111/cns.14414 (PMC10805448; doi:10.1111/cns.14414)
Supplement: Supplementary file 1 — Figure S1. Figure S2. [file CNS-30-e14414-s001.docx]

**Figure S1** showed the degree of the whole brain in TLE group, and **Figure S2** showed the degree of the whole brain in NC group. Further, degrees of 90 cortex regions in groups of TLE and NC listed in descending order. The red areas were regarded as hubs (degree > group mean + SD). Classification of brain regions was based on AAL proposed by Tzourio-Mazoyer (Tzourio-Mazoyer et al, 2002). TLE, temporal lobe epilepsy; NC, normal controls; L, left; R, right
